# Lenalidomide Polarizes Th1-specific Anti-tumor Immune Response and Expands XBP1 Antigen-Specific Central Memory CD3^+^CD8^+^ T cells against Various Solid Tumors

**DOI:** 10.4172/2329-6917.1000178

**Published:** 2015-04-21

**Authors:** Jooeun Bae, Derin B Keskin, Kristen Cowens, Ann-Hwee Lee, Glen Dranoff, Nikhil C Munshi, Kenneth C Anderson

**Affiliations:** 1 Dana-Farber Cancer Institute, Boston, Massachusetts, USA; 2 Harvard Medical School, Boston, Massachusetts, USA; 3 Weill Cornell Medical College, New York, NY, USA; 4 VA Boston Healthcare System, Boston, Massachusetts, USA

**Keywords:** Combination immunotherapy, Lenalidomide, XBP1, Antigen-specific cytotoxic T lymphocytes, Cancer vaccine

## Abstract

**Introduction:**

Effective combination immunotherapeutic strategies may be required to enhance effector cells’ anti-tumor activities and improve clinical outcomes.

**Methods:**

XBP1 antigen-specific cytotoxic T lymphocytes (XBP1-CTL) generated using immunogenic heteroclitic XBP1 US184-192 (YISPWILAV) and XBP1 SP367-375 (YLFPQLISV) peptides or various solid tumor cells over-expressing XBP1 target antigen were evaluated, either alone or in combination with lenalidomide, for phenotype and immune functional activity.

**Results:**

Lenalidomide treatment of XBP1-CTL increased the proportion of CD45RO^+^ memory CD3+CD8+ T cells, but not the total CD3^+^CD8^+^ T cells. Lenalidomide upregulated critical T cell activation markers and costimulatory molecules (CD28, CD38, CD40L, CD69, ICOS), especially within the central memory CTL subset of XBP1-CTL, while decreasing TCRαβ and T cell checkpoint blockade (CTLA-4, PD-1). Lenalidomide increased the anti-tumor activities of XBP1-CTL memory subsets, which were associated with expression of Th1 transcriptional regulators (T-bet, Eomes) and Akt activation, thereby resulting in enhanced IFN-γ production, granzyme B upregulation and specific CD28/CD38-positive and CTLA-4/PD-1-negative cell proliferation.

**Conclusions:**

These studies suggest the potential benefit of lenalidomide treatment to boost anti-tumor activities of XBP1-specific CTL against a variety of solid tumors and enhance response to an XBP1-directing cancer vaccine regime.

## Introduction

Active-specific immunotherapy has the goal of inducing effector T lymphocytes with anti-tumor activities, durable immune responses with favorable side effect profile, and long-term stabilization of disease. To accomplish the goal, immunotherapy must address the tolerogenic mechanisms deployed by tumors as well as immunosuppression in cancer patients due to impaired antigen-processing ability, downregulation of MHC class I, and increased levels of immune-suppressive cells and cytokines. Tumors often produce excessive amounts of immunosuppressive mediators such as adenosine, kynurenines, indoleamine 2,3-dixoxygenase, prostaglandin E2, TGF-β and VEGF-α [1–3]. Hence, disease-related immunosuppression is an important hurdle to overcome for immunotherapeutic strategies to be successful. Approaches including adjuvant selection have been developed to both enhance anti-tumor activities and overcome the suppressive effects within the tumor microenviroment.

Lenalidomide is an immunomodulatory drug (IMiD) with potential immune stimulatory anti-angiogenic and direct anti-tumor effects, which together act against tumor in its microenvieonment [4–6]. Studies on effector cells have found that IMiDs enhance cross-priming of naive CD8^+^ T cells by dendritic cells, activate adaptive T-cell immunity via activation of the CD28 signaling pathway, and polarize T-cell immunity toward Th1-specific response. Importantly, lenalidomide has been shown to suppress regulatory T cells, T-cell expression of CTLA-4 and PD-1, and restore T-cell immunologic synapse dysfunction [7–9]. IMiDs also inhibit the production of TNF-α and enhance innate immune responses in the bone marrow microenvironment in multiple myeloma (MM) by overcoming suppression of IFN-γ/IL-2 cytokine signaling in immune effector cells [10–12]. These findings suggest that IMiD may provide a potential adjuvant function in cancer vaccine immunotherapy. However, the ability of an IMiD to enhance or modify tumor-specific immunity in tumor directed vaccine derived CTL effector cells has not been fully explored.

XBP1 is an important transcription activator of the unfolded protein response (UPR), while activates a distinct set of genes and regulates endoplasmic reticulum (ER) stress-mediated apoptosis [13]. Previously, XBP1 has been identified as a therapeutic target in MM due to its’ differential expression between plasma cells from normal donors and patients’ with monoclonal gammopathy of undetermined significance (MGUS) or MM, as well as its uniform expression in all MM patient cells and cell lines [14–17]. XBP1 has also been implicated in the proliferation of malignant plasma cells. Moreover, studies have found that XBP1 is overexpressed in a variety of solid tumors by various analyses, i.e. genome-wide profiling, flow cytometry, and immunohistochemistry; and is required for functions including tumor cell growth and survival and proliferation under hypoxic conditions [18–21]. Currently, several agents are in development to target XBP1 including Irestatin, an inhibitor of IRE1 and the UPR through inhibition of spliced XBP1 transcription activity; and Trierixin, a new member of the triene-ansamycin group which is a novel inhibitor of ER-stress induced cleavage of XBP1[22,23].

Our study examines a new selective immunotherapeutic strategy by targeting XBP1 in different types of cancers including MM [24,25], SMM [26], breast cancer, colon cancer, and pancreatic cancer [18]. Our previous studies have shown the immunotherapeutic potential of heteroclitic XBP1 US_184-192_ (YISPWILAV) and XBP1 SP_367-375_ (YLFPQLISV) peptides to generate XBP1 antigen-specific CTL, and we demonstrate here that lenalidomide can enhance anti-tumor activities of XBP1-CTL against a variety of solid tumors which overexpress XBP1 antigen.

## Material and Methods

### Cell lines

Breast cancer (MDA-MB231, MCF-7) and colon cancer (LS180, SW480) cell lines were obtained from the ATCC (Manassas, VA). The pancreatic cancer cell lines (PATU8988T, Panc1) were kindly provided by Dr. A. Kimmelman (Harvard Medical School, Boston, MA). The T2 cell line was provided by Dr. J. Molldrem (University of Texas M. D. Anderson Cancer Center, Houston, TX). MDA-MB231 and SW480 cell lines were cultured in Leibovitz’s L-15 Medium (ATCC); MCF-7 and LS180 cell lines were cultured in EMEM (Gibco-Life Technologies, Rockville, MD); and Panc1, PATU8902, and T2 cell lines were cultured in DMEM (Gibco-Life Technologies) media. All media were supplemented with 10% fetal calf serum (FCS; BioWhittaker, Walkersville, MD), 100 IU/ml penicillin, and 100 μg/ml streptomycin (Gibco-Life Technologies).

### Synthetic peptides

HLA-A2-specific immunogenic heteroclitic XBP1_184-192_ (YISPWILAV) and heteroclitic XBP1 SP_367-375_ (YLFPQLISV) peptides were synthesized by standard fmoc (9-fluorenylmethyl-oxycarbonyl) chemistry, purified to >95% using reverse-phase chromatography, and validated by mass-spectrometry for molecular weight (Biosynthesis, Lewisville, TX). Lyophilized peptides were dissolved in DMSO (Sigma, St. Louis, MO), diluted in AIM-V medium (Gibco-Life Technologies), and stored at −140°C until needed.

### Reagents

Fluorochrome conjugated anti-human mAb specific to HLA-A2, CD3, CD8, CD28, CD38, CD40L, CD69, ICOS, TCRαβ, CCR7, CD45RO, CD80, CD86, ICOS ligand, IFN-γ, CTLA-4, PD-1, PD-L1, PD-L2, T-bet, granzyme B or Akt were purchased from Becton Dickinson (San Diego, CA). Fluorochrome conjugated anti-human Eomes mAb was purchased from eBioscience (San Diego, CA). Recombinant human IL-2, IL-4, IFN-α and TNF-α were purchased from R&D Systems, and human GM-CSF was obtained from Immunex (Seattle, WA). Lenalidomide (Celgene, Summit, NJ) was reconstituted in 1% DMSO and was used at a final concentration of 5 μM to treat either XBP1-CTL or various tumor cell lines.

### Generation of XBP1-CTL and treatment with lenalidomide

XBP1 peptides-specific CTL (XBP1-CTL) were generated *ex vivo* by repeated stimulation of CD3^+^ T lymphocytes obtained from HLA-A2^+^ normal donors with a cocktail of immunogenic heteroclitic XBP1 peptides-pulsed antigen-presenting cells (APC). In brief, APC in serum-free AIM-V medium were pulsed overnight at 37°C and 5% CO^2^ in humidified air with a cocktail (50 μg/ml) of heteroclitic XBP1 US_184-192_ (YISPWILAV) and heteroclitic XBP1 SP_367-375_ (YLFPQLISV) peptides. The peptides pulsed APC were washed, irradiated, and used to prime CD3^+^ T cells at a 1:20 APC/peptide (stimulator)-to-CD3^+^ T cell (responder) ratio in AIM-V medium supplemented with 10% human AB serum (BioWhittaker) and IL-2 (50 U/ml). The CTL cultures were restimulated with the heteroclitic XBP1 peptide pulsed-APC every seven days for a total of 4 cycles. After the last stimulation, XBP1-CTL were treated with lenalidomide (5 μM) for 4 days and evaluated for their phenotype and functional activities. XBP1-CTL cultured in the presence of DMSO (1% final concentration) for 4 days were used as a comparative control in these studies.

### Evaluation of the effects of lenalidomide on expression of critical T cell markers on CD3^+^CD8^+^ T cells or on different T cell subtypes of XBP1-CTL

XBP1-CTL were evaluated for the frequency of CD3^+^CD8^+^ T cells and expression levels (% positive cells, mean fluorescence intensity (MFI)) of critical T cell surface markers including CD45RO, CCR7, CD28, CD38, CD40L, CD69, ICOS, TCRαβ, CTLA and PD-1. After staining with each specific antibody, the cells were washed and fixed in 2% paraformaldehyde. The cells were analyzed using a LSRII Fortessa^™^ flow cytometer and DIVA^™^ v8.0 software (BD). The XBP1-CTL were gated on non-memory or memory CD3^+^CD8^+^ T cells and central memory (CM), effector memory (EM) or terminal effector (TE) CD3^+^CD8^+^ T cell subsets.

### Analysis of lenalidomide effects on the expression of surface proteins or intracellular proteins on cancer cell lines

Breast cancer (MDA-MB231, MCF-7), colon cancer (LS180, SW480), and pancreatic cancer (PATU8902, Panc1) cell lines were treated with lenalidomide (5 μm in DMSO, Celgene) for 4 days. As controls, each of the cancer cell lines was cultured in the presence of DMSO (1% final concentration) for 4 days. The cancer cell lines were evaluated with the treatment for their phenotype changes of surface markers including HLA-A2, CD80, CD86, ICOS ligand, PD-L1 and PD-L2. Separately, the lenalidomide or DMSO treated cancer cells were evaluated for intracellular expression of XBP1 unspliced or XBP1 spliced protein. In brief, each of the cancer cell lines were fixed and permeabilized using the Cytofix/Cytoperm kit (BD) and stained with rabbit anti-human XBP1 unspliced isoform monoclonal antibody (mAb) (Novus Biologicals, Littleton, CO) or mouse anti-human XBP1 spliced isoform mAb (R&D Systems, Minneapolis, MN) for 30 minutes at room temperature, washed with Perm/Wash solution (BD) and stained with donkey anti-rabbit IgG-PE (Novus Biologicals, Littleton, CO) or goat anti-mouse IgG-PE (R&D Systems, Minneapolis, MN), respectively, for 30 minutes at 4°C. The cells were washed with Perm/Wash solution and fixed in 2% formaldehyde-PBS. After staining with each specific antibody, the tumor cells were washed and analyzed using a LSRII Fortessa^™^ flow cytometer and DIVA^™^ v8.0 software (BD).

### Examination of lenalidomide effects on the expression of T-bet, Eomes and Akt and anti-tumor functional activities of XBP1-CTL

The expression of transcriptional regulators and signal integrator or tumor-specific responses were evaluated in XBP1-CTL upon lenalidomide treatment. In brief, XBP1-CTL were co-incubated with each cancer cell line for 6 hours, and they were washed and stained with fluorochrome conjugated mAbs specific to surface antigens including CD3, CD8, CD45RO, and CCR7. They were further fixed and permeabilized, and stained with mAbs specific to IFN-γ, granzyme B, T-bet, Eomes and/or AKT. The cells were analyzed using a LSR Fortessa^™^ flow cytometer and DIVA^™^ v8.0 software after gating on non-memory or memory CD3^+^CD8^+^ CTL populations.

### Analysis of lenalidomide effects on XBP1-CTL proliferation in response to tumor cell lines

To evaluate tumor-specific CTL proliferation, CFSE (Molecular Probes, Eugene, OR) labeled XBP1-CTLwere co-incubated with irradiated (20 Gy) cancer cell lines. On day 6, the cultures were harvested, stained with anti-CD3,CD8, CD45RO, CCR7, CD28, CD38, CTLA-4 and/or PD-1 fluorochrome conjugated mAbs, and analyzed by flow cytometry to determine their specific cell proliferation.

### Statistical analysis

Results are presented as mean } SE. Groups were compared using unpaired Student’s t-test. Differences were considered significant when *p<0.05.

## Results

### Lenalidomide increases the frequency of memory CD8^+^ T cell subsets of XBP1 antigen-specific CTL

**Figure 1:**
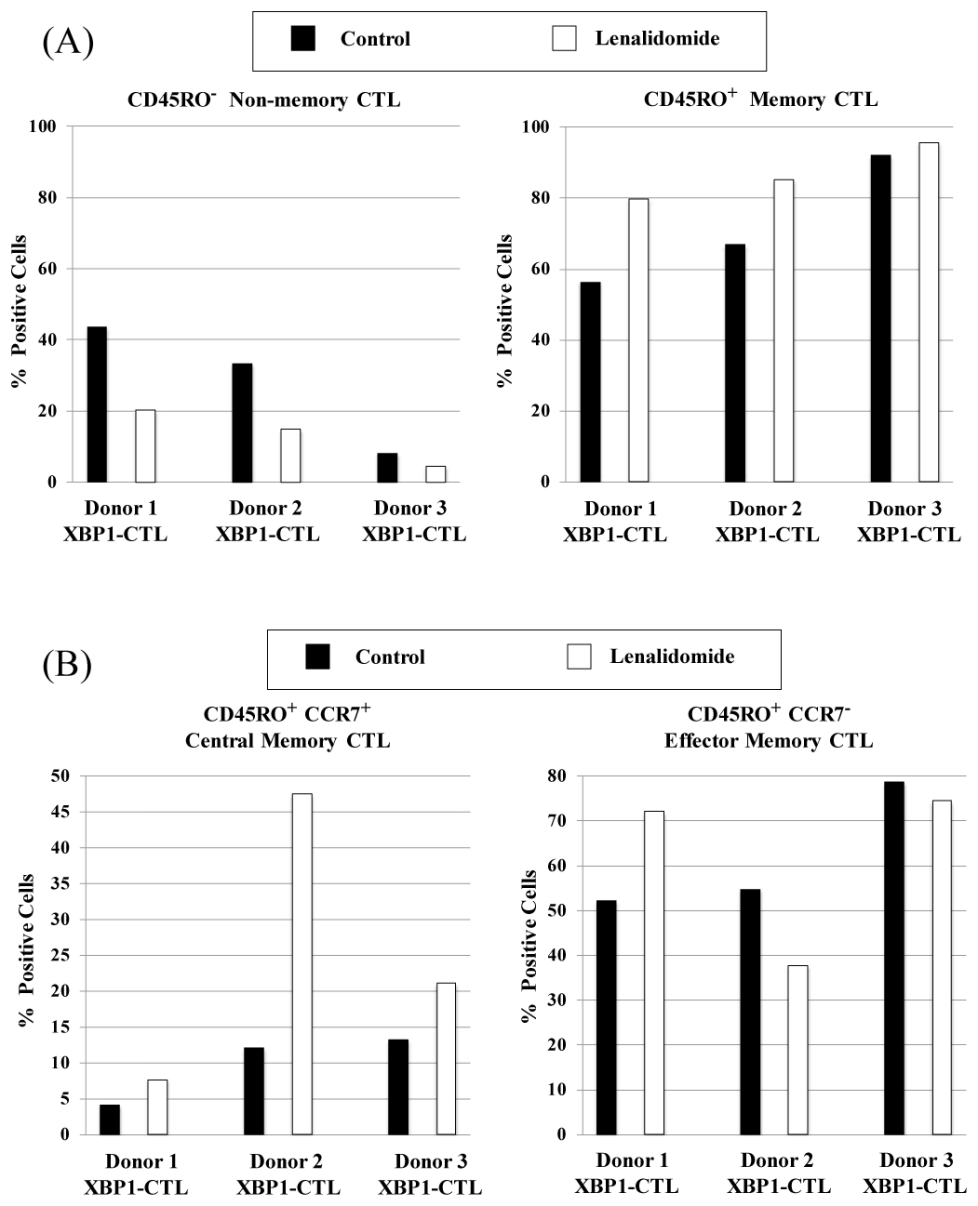
Lenalidomide induced CD3^+^CD8^+^ T cell memory subsets in XBP1-CTL. XBP1-CTL were generated from three respective HLA-A2^+^ normal donors’ CD3^+^ T cells by repeated stimulation, once a week, with a cocktail of heteroclitic unspliced XBP1_184-192_ (YISPWILAV) and spliced XBP1 SP_196-204_ (YLFPQLISV) peptides (50 μg/ml; 25 μg/peptide). After the 4th stimulation, XBP1-CTL were treated with lenalidomide (5 μm, 4 days) and examined for their phenotypic T cell profile by flow cytometry. (A) Lenalidomide treatment induced a higher frequency of memory CTL (CD45RO^+^/CD3^+^CD8^+^) and a corresponding lower frequency of non-memory CTL (CD45RO^−^/CD3^+^CD8^+^) as compared to the control XBP1-CTL. (B) Lenalidomide treatment increased the percentage of central memory (CD45RO^+^CCR7^+^) CD3^+^CD8^+^ T cell subset within the memory cells of all the XBP1-CTL generated (n=3).

XBP1-CTL generated using immunogenic heteroclitic XBP1 US_184-192_ (YISPWILAV) and XBP1 SP_367-375_ (YLFPQLISV) peptides were phenotypically evaluated, with or without treatment with Lenalidomide (5 μm, 4 days). Lenalidomide did not induce a change in the total CD3^+^CD8^+^ T cell frequency of XBP1-CTL, nor in cell viability (data not shown). However, an increase of CD45RO^+^ memory CD3^+^ CD8^+^ T cells and corresponding decrease of CD45RO^−^ non-memory CD3^+^ CD8^+^ T cells were observed after lenalidomide treatment in the XBP1-CTL, as compared to control XBP1-CTL (Figure 1A). The effect of lenalidomide on XBP1-CTL memory CD3^+^CD8^+^ T cells was further investigated. As compared to the EM (CD45RO^+^ CCR7^−^) subset, the CM (CD45RO^+^ CCR7^+^) subset frequency was consistently increased in XBP1-CTL (Donor 1: 95% increase, Donor 2: 292% increase, Donor 3: 50% increase), after treatment with lenalidomide (Figure 1B).

### Lenalidomide regulates key activating and inhibitory molecules on XBP1-CTL

**Figure 2:**
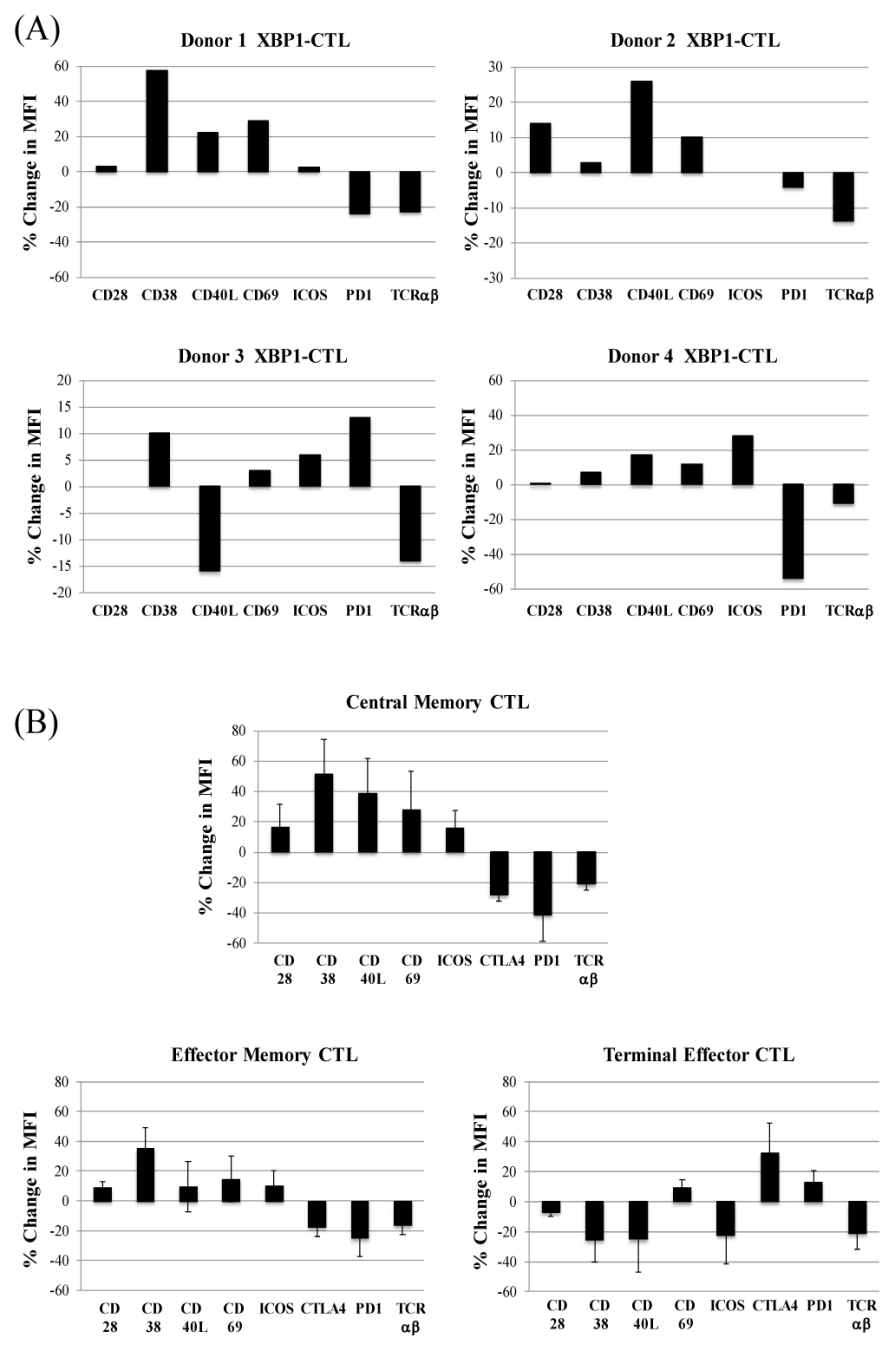
Lenalidomide changed T cell antigen expression on XBP1-CTL with primary modification in the central memory CD3^+^CD8^+^ T cell subset XBP1-CTL (n=4) were evaluated after lenalidomide (5 μm, 4 days) treatment for changes in expression of critical T cell activation markers (CD38, CD69), co-stimulatory molecules (CD28, CD40L, ICOS), inhibitory molecule (PD-1, CTLA-4) and T-cell receptor (TCRαβ). Results obtained by flow cytometric analyses are presented as the ‘% Change in MFI (mean fluorescence intensity)’, which was calculated as [(MFI Lenalidomide treated XBP1-CTL/MFI Control XBP1-CTL) x100]’ for each specific marker. (A) Lenalidomide upregulated the T cell activation markers (CD38, CD69: 4/4) and co-stimulatory molecules (CD28: 2/4, CD40L: 3/4, ICOS: 3/4) on XBP1-CTL, while down-regulating inhibitory molecule (PD-1: 3/4) and T-cell receptor (TCRαβ: 4/4). (B) The upregulation of T cell activation markers (CD38, CD69) and costimulatory molecules (CD28, CD40L, ICOS) and downregulation of inhibitory molecules (PD-1, CTLA-4) and T-cell receptor (TCRαβ) following lenalidomide treatment were mainly detected within the central memory (CD45RO^+^CCR7^+^) CD3^+^CD8^+^ T cell subset of XBP1-CTL.

The effect of lenalidomide was next evaluated on expression of key molecules for T cell co-stimulation and cellular activation (CD28, CD38, CD40L, CD69, ICOS) and T cell inhibition (PD-1) or TCRαβ on the XBP1-CTL. Lenalidomide induced upregulation of CD28 (2/4 donors), CD38 (4/4 donors), CD40L (3/4 donors), CD69 (4/4 donors), and ICOS (3/4 donors) on the XBP1-CTL generated from different HLA-A2^+^ donors (Figure 2A). In contrast, lenalidomide reduced the expression levels of PD-1 (3/4 donors) and TCRαβ (4/4 donors). XBP1-CTL phenotypic changes following lenalidomide treatment were further evaluated within specific memory or non-memory CD3^+^ CD8^+^ T cell subsets. Lenalidomide treated XBP1-CTL displayed increased levels of cellular activation, evaluated by upregulation of both CD38 and CD69. Interestingly, the central memory (CD45RO^+^ CCR7^+^) CTL subset, which is the main population increased by lenalidomide, also showed high upregulation of molecules involved in T cell activation/costimulation (Figure 2B). The terminal effector (CD45RO^−^ CCR7^−^) CTL subset showed downregulation of the CD38 cellular activation marker and decreased expression of CD28, CD40L, and ICOS post lenalidomide treatment. Importantly, the expression of T-cell inhibitory molecules, CTLA-4 and PD-1, was reduced on all XBP1-CTL memory subsets (CM. EM) after lenalidomide treatment, while their expression was increased on the non-memory subset (TE) (Figure 2B). Finally, lenalidomide consistently downregulated TCRαβ expression in all subsets of XBP1-CTL (Figures 2A and 2B).

### Lenalidomide does not modify the expression of intracellular XBP1 proteins nor critical co-stimulatory or inhibitory surface antigens on breast cancer, colon cancer and pancreatic cancer cells

**Figure 3:**
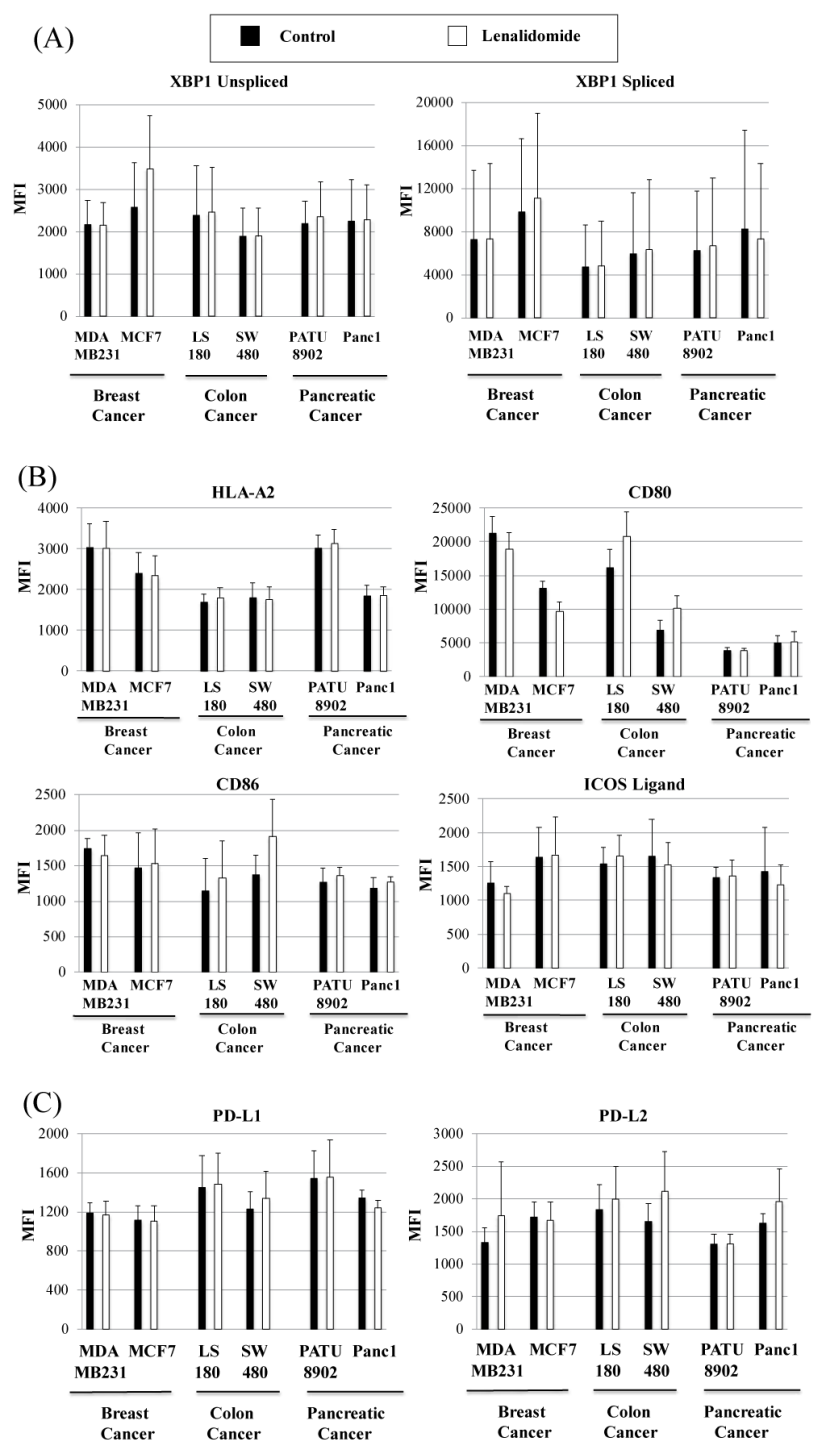
Lenalidomide treatment does not affect expression of intracellular XBP1 proteins and surface antigens on breast, colon and pancreatic cancer cells The effect of lenalidomide (5 μm, 4 days) was evaluated on the antigen expression of various cancer cells; breast cancer (MDA-MB231, MCF7), colon cancer (LS180, SW480), pancreatic cancer (PATU8902, Panc1). No major changes were detected in the expression levels of (A) intracellular XBP1 unspliced and XBP1 spliced proteins, (B) surface HLA-A2, CD80, CD86 and ICOS ligand, nor (C) surface PD-L1 and PD-L2. Results were obtained by flow cytometry analyses and shown as the MFI (mean fluorescence intensity; n=3, mean ± SE. Groups were compared using an unpaired Student’s t-test and no differences (p > 0.05) were seen in MFI between control and lenalidomide treated tumor cells.

In contrast to its effects on antigen-specific CTL, lenalidomide treatment (5 μm, 4 days) did not significantly (p>0.05) change the expression of intracellular XBP1 unspliced and XBP1 spliced target antigens in breast cancer (MDA-MB231, MCF7), colon cancer (LS180, SW480) and pancreatic cancer (PATU8902, Panc1) (Figure 3A). Further analyses were performed to investigate modification of costimulatory and inhibitory surface antigens on these tumor cells. Our results showed that lenalidomide treatment did not modify expression (p>0.05) of HLA-A2, a MHC class I molecule having affinity for endogenous XBP1 peptides. In addition, the expression levels of CD80 and CD86 (the B7 co-stimulatory molecules) and ICOS-L, which is the ligand for the T-cell-specific cell surface receptor ICOS and acts as a costimulatory signal for T-cell proliferation and cytokine secretion, were not changed (p>0.05) on HLA-A2^+^ breast cancer, colon cancer and pancreatic cancer cell lines after lenalidomide treatment (Figure 3B). Similarly, no drug-related change (p>0.05) was noted in expression of the inhibitory program-death ligands PD-L1 or PD-L2 on tumor cells (Figure 3C).

### Lenalidomide increases IFN-γ production and granzyme B upregulation by memory CD3^+^CD8^+^ T cells of XBP1-CTL in response to various solid tumor cells

**Figure 4:**
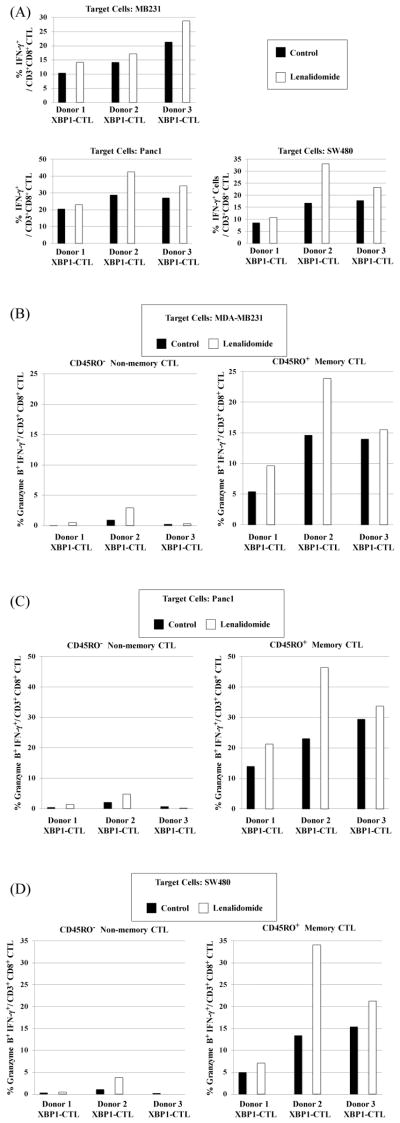
Lenalidomide enhanced anti-tumor activities of XBP1-CTL memory cells against solid tumor cells XBP1-CTL (n=3) treated with lenalidomide (5 μm, 4 days) were analyzed for their functional activities against HLA-A2^+^ breast cancer cell (MDA-MB231), pancreatic cancer cell (Panc1) or colon cancer (SW480) cells. (A) Lenalidomide increased IFN-γ production by total CD3^+^CD8^+^ T cells in response to the respective cancer cells. High levels of granzyme B upregulation and IFN-γ production were detected within the CD45RO^+^ memory CTL population in response to (B) MDA-MB231, (C) Panc1 or (D) SW480 tumor cells. Results are expressed as ‘% positive cells’ of the specific CD3^+^CD8^+^ T cell memory subsets for either single or poly-functional anti-tumor activity.

In our previous studies, XBP1-CTL induced using the HLA-A2-specific XBP1 US_184-192_ (YISPWILAV) and XBP1 SP_367-375_ (YLFPQLISV) peptides displayed HLA-A2-restricted and antigen-specific activities against breast cancer, colon cancer and pancreatic cancer cells [18]. In current studies, we further evaluated XBP1-CTL post-lenalidomide treatment for their specific anti-tumor activity by measuring IFN-γ production and granzyme B expression against the respective HLA-A2^+^ solid tumor cells. As compared to the XBP1-CTL control, lenalidomide-treated XBP1-CTL (n=3) showed increased IFN-γ production in response to HLA-A2^+^ MDA-MB231 (breast cancer), Panc1 (pancreatic cancer) or SW480 (colon cancer) cells (Figure 4A). The anti-tumor response of XBP1-CTL was further investigated within the CD45RO^+^ memory or CD45RO^−^ non-memory CD3^+^CD8^+^ T cell populations in response to the respective tumor cell lines. Compared to non-memory cells, the memory cells of XBP1-CTL demonstrated significantly greater anti-tumor activities, measured by granzyme B upregulation and IFN-γ production. The activities of different memory cell subsets were specifically enhanced after lenalidomide treatment in XBP1-CTL (n=3) against breast cancer (MDA-MB231; Figure 4B), pancreatic cancer (Panc1; Figure 4C) and colon cancer (SW480; Figure 4D) cells.

### Lenalidomide enhances tumor-specific central memory cell proliferation within XBP1-CTL

**Figure 5:**
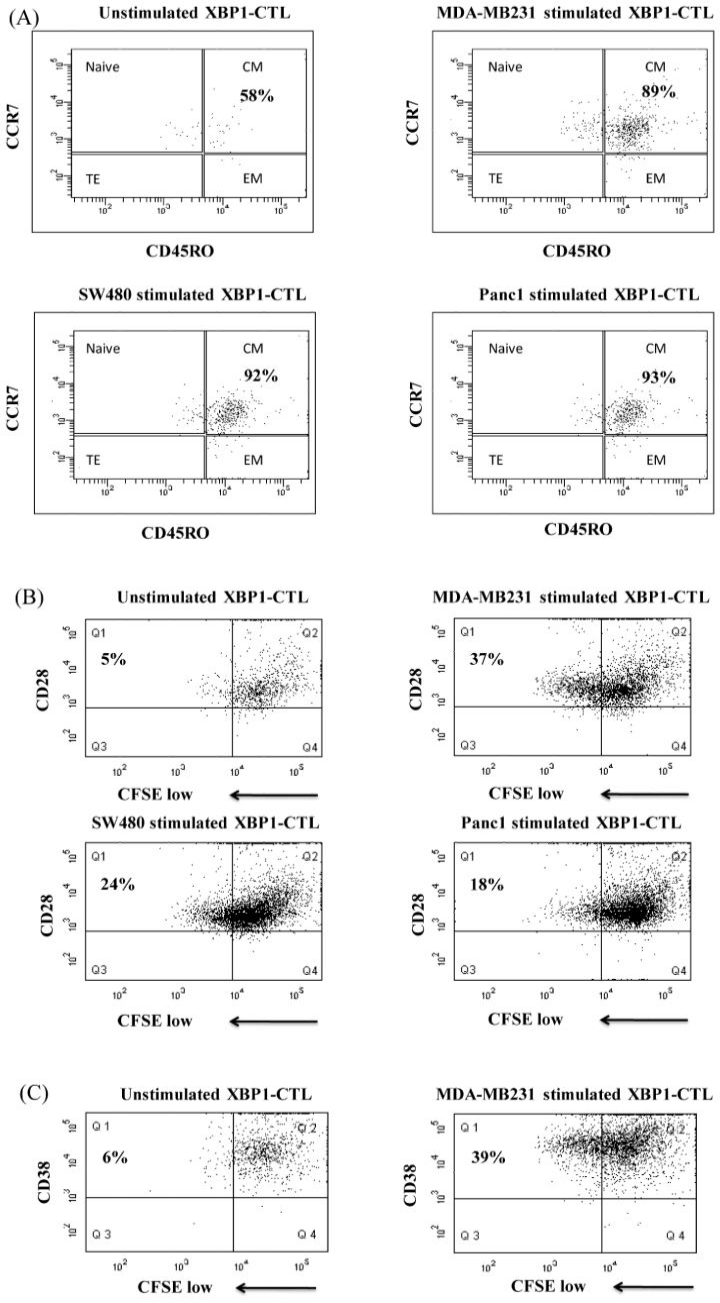

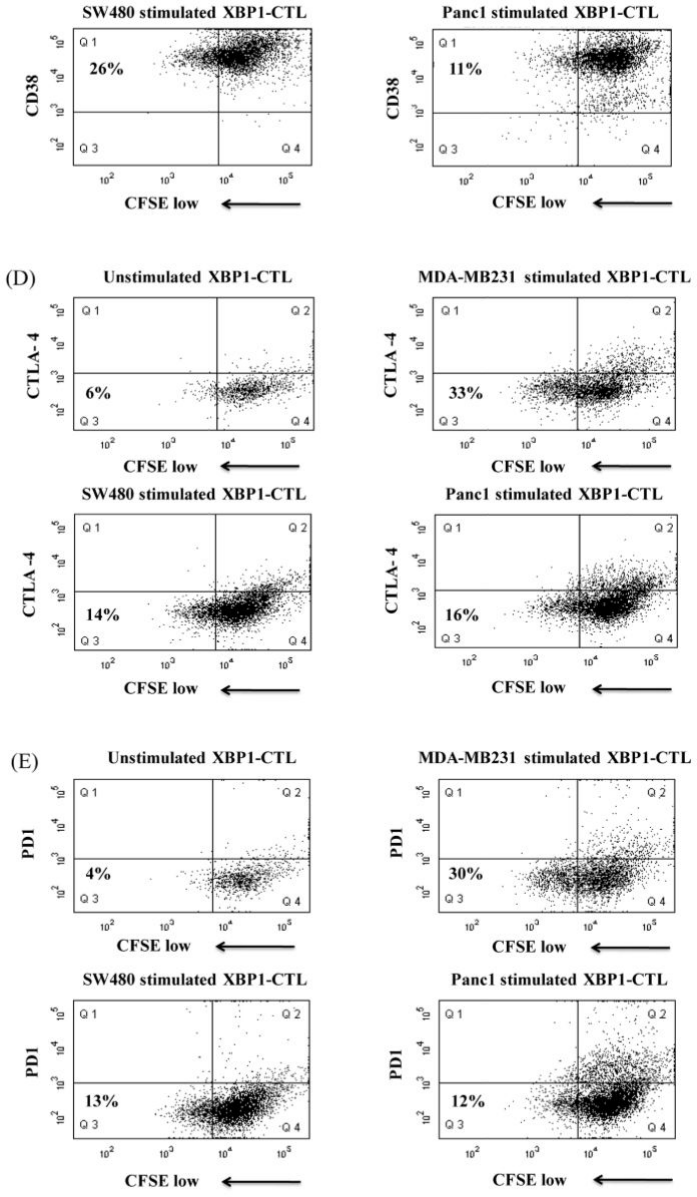
Lenalidomide increased specific XBP1-CTL proliferation to breast cancer, colon cancer or pancreatic cancer cells Specific cell proliferation was examined by flow cytometry after stimulating CFSE-labeled XBP1-CTL with tumor cells for 6 days. The proliferating CD3^+^CD8^+^T cell population was measured as the percent decrease in CFSE expression. Enhanced cell proliferation of lenalidomide treated (5 μm, 4 days) XBP1-CTL was examined in response to irradiated respective tumor cells. Background proliferation was determined using CFSE-labeled lenalidomide treated XBP1-CTL cultured in media alone. (A) The major proliferative response of XBP1-CTL after lenalidomide treatment was detected within the central memory (CCR7^+^CD45RO^+^) CTL subset against breast cancer (MDA-MB231), colon cancer (SW480) or pancreatic cancer (Panc1) cells. Phenotypic characterization demonstrates that specific cellular proliferation (Q1 gated) to HLA-A2+ breast cancer (MDA-MB231), colon cancer (SW480) or pancreatic cancer (Panc1) cells express (B) CD28 and (C) CD38, but neither (D) CTLA-4 nor (E) PD-1.

Previously, we have shown that memory CD3^+^CD8^+^ T cells are the major cell subset within XBP1-CTL that proliferate in response to various solid tumor cells [18]. Here, we further evaluated the effect of lenalidomide on XBP1-CTL proliferative capacity in CFSE-based assays. First, anti-tumor specific cell proliferation was measured by gating on the CFSE-low CD3^+^CD8^+^ T cell population, and then analyzing for the specific naïve:memory CTL subset involved. Lenalidomide treated XBP1-CTL showed a distinctive cell proliferation pattern, while was restricted mainly to the central memory (CD45RO^+^CCR7^+^) CD3^+^CD8^+^ T cell subset, in response to the respective solid tumor cells including HLA-A2^+^ breast cancer (MDA-MB231; 89% CM proliferation), colon cancer (SW480; 92% CM proliferation) or pancreatic cancer (Panc1; 93% CM proliferation) cells (Figure 5A). Upon identification of the CM subset as the predominant proliferating population, we next investigated the specific phenotypic cell populations proliferating within the XBP1-CTL. These analyses revealed higher proliferation (Q1) of CD28-positive (Figure 5B) and CD38-positive (Figure 5C) cells within XBP1-CTL by lenalidomide treatment against breast cancer (MDA-MB231; 37% CD28^+^, 39% CD38^+^ proliferation), colon cancer (SW480; 24% CD28^+^, 26% CD38^+^ proliferation) or pancreatic cancer (Panc1; 18% CD28^+^, 11% CD38^+^ proliferation) cells. In contrast, control XBP1-CTL did not show any specific cell proliferation in the absence of tumor cell stimulation (Figure 5B; 5% CD28^+^ proliferation, Figure 5C; 6% CD38^+^ proliferation). Furthermore, lenalidomide did not trigger proliferation (Q1) of CTL expressing immune checkpoint inhibitors CTLA-4 (Figure 5D) or PD-1 (Figure 5E) in response to the various HLA-A2^+^ tumor cells. XBP1-CTL proliferation after lenalidomide treatment was mainly restricted to CTLA-4 negative (Q3, Figure 5D) or PD-1 negative (Q3, Figure 5E) CD3^+^CD8^+^ T cells. These responses were consistent against the various solid tumors including MDA-MB231 breast cancer cells (33% CTLA-4^−^ proliferation, 30% PD1^−^ proliferation), SW480 colon cancer cells (14% CTLA-4^−^ proliferation, 13% PD1^−^ proliferation) or Panc1 pancreatic cancer cells (16% CTLA-4^−^ proliferation, 12% PD1^−^ proliferation).

### Lenalidomide increases T-bet expression in the XBP1-CTL, along with IFN-γ production, in response to solid tumor cells

**Figure 6:**
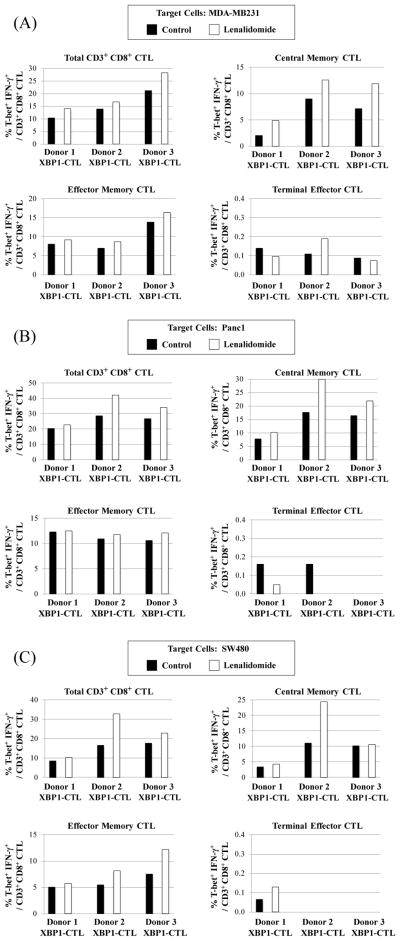
Lenalidomide enhanced T-bet upregulation and IFN-γ production by XBP1-CTL in response to breast cancer, colon cancer or pancreatic cancer cells Lenalidomide treated XBP1-CTL (n=3) were evaluated for T-bet expression and IFN-γ production in response to a variety of solid tumor cells by flow cytometric analyses. Lenalidomide increased the frequency of Tbet^+^/IFN-γ+ cells within the total CD3^+^CD8^+^ T cells or specific central (CM) and effector memory (EM) cell subsets, but not the terminal effector (TE) cell subset, by stimulation of XBP1-CTL with (A) breast cancer cells (MDA-MB231), (B) pancreatic cancer cells (Panc1) or (C) colon cancer cells (SW480).

T-bet is an important transcription regulator which promotes the generation and function of effector and memory CD8^+^ T cells. In previous studies, we have shown that XBP1 antigen-specific CTL generated with a cocktail of HLA-A2-specific heteroclitic XBP1 US_184-192_ (YISPWILAV) and XBP1 SP_367-375_ (YLFPQLISV) peptides express high levels of T-bet [18]. In these studies, we further evaluated the effect of lenalidomide treatment on XBP1-CTL (n=3) functional activities associated with its T-bet expression and function, in response to each type of solid tumor cells. Our results showed that the frequency of T-bet expressing and IFN-γ producing XBP1-CTL was consistently higher in total CD3^+^CD8^+^ T cells after lenalidamide treatment, as compared to untreated XBP1-CTL in response to breast cancer (MDA-MB231, Figure 6A), pancreatic cancer (Panc1, Figure 6B) or colon cancer (SW480, Figure 6C) cells. Importantly, the T-bet^+^ IFN-γ^+^ cells observed within the lenalidomide treated XBP1-CTL were confined to memory CD3^+^CD8^+^ T cells, with the greatest increase within the central memory CTL subset (Figures 6A and 6B). In contrast, lenalidomide had no affect on the frequency of T-bet^+^ IFN-γ^+^ cells within the terminal effector CTL population (Figures 6A–6C).

### Lenalidomide increases Eomes expression in the XBP1-CTL, along with IFN-γ production, in response to solid tumor cells

**Figure 7:**
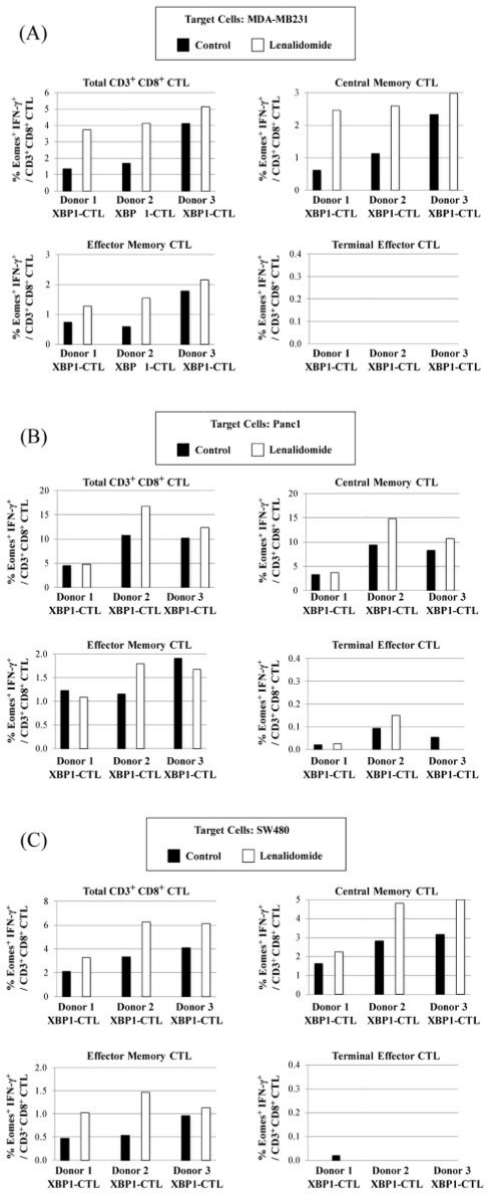
Lenalidomide enhanced Eomes upregulation and IFN-γ production by XBP1-CTL in response to breast cancer, colon cancer or pancreatic cancer cells Lenalidomide treated XBP1-CTL (n=3) were evaluated for Eomes expression and IFN-γ production in response to a variety of solid tumor cells by flow cytometric analyses. Lenalidomide increased the frequency of Eomes^+^/IFN-γ^+^ cells within the total CD3^+^CD8^+^ T cells or specific central (CM) and effector memory (EM) cell subsets, but not the terminal effector (TE) cell subset, by stimulation of XBP1-CTL with (A) breast cancer cells (MDA-MB231), (B) pancreatic cancer cells (Panc1) or (C) colon cancer cells (SW480).

Next, the effect of lenalidomide was evaluated on Eomes, another important transcription factor, regulating memory CTL generation. Consistent with T-bet, we observed upregulation of Eomes expression following lenalidomide treatment of XBP1-CTL. In addition, the frequency of Eomes^+^ IFN-γ^+^/CD3^+^ CD8^+^ T cells was increased in response to breast cancer (MDA-MB231, Figure 7A), pancreatic cancer (Panc1, Figure 7B) or colon cancer (SW480, Figure 7C) cells in the lenalidomide treated XBP1-CTL. In conjunction with the T-bet^+^ CTL and their activities, we detected an increase in the frequency of Eomes^+^ IFN-γ^+^ cells within the central memory CD3^+^CD8^+^ T cell subset after lenalidomide treatment (Figures 7A–C). Finally, no or very low levels of Eomes^+^IFN-γ^+^/CD3^+^ CD8^+^ T cells were observed within the terminal effector subset of lenalidomide treated XBP1-CTL. Thereby, these studies demonstrate the critical immune modulatory activity of lenalidomide on memory CTL subsets within antigen-specific effector cells.

### Lenalidomide increases the serine/threonine-specific protein kinase AKT expression in memory subsets of XBP1-CTL

**Figure 8:**
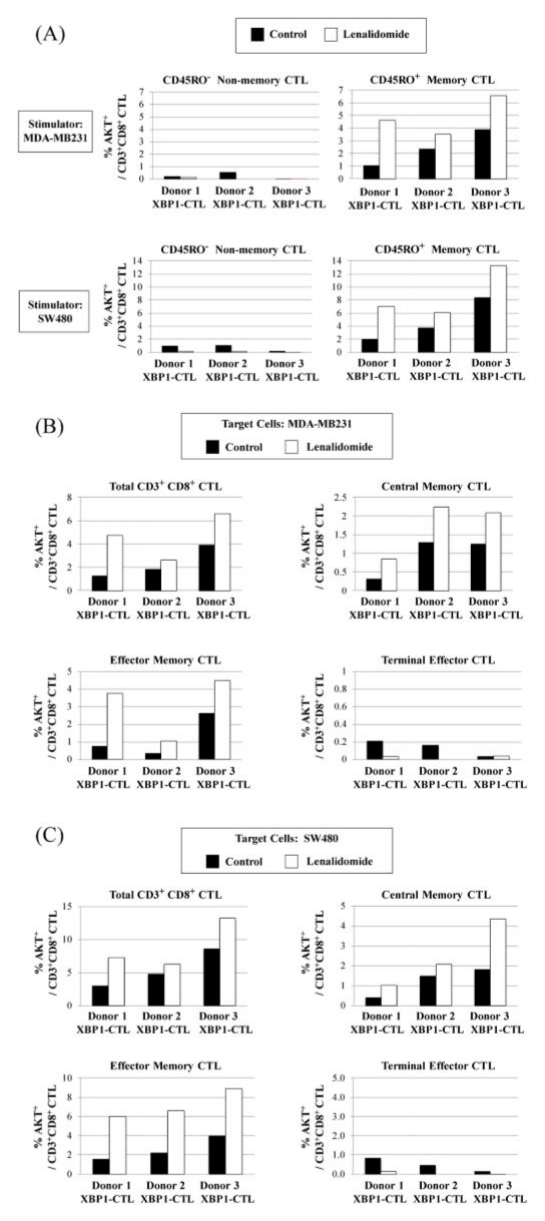
Lenalidomide enhanced Akt activation in memory subsets of XBP1-CTL in response to tumor cells Lenalidomide treated XBP1-CTL (n=3) were evaluated for Akt activation upon stimulation by flow cytometry analyses. (A) Lenalidomide treated XBP1-CTL (n=3) showed the upregulation of Akt in the CD45RO^+^ memory CD3^+^CD8^+^ T cell population, but not in the CD45RO^−^ non-memory CD3^+^CD8^+^ T cell population, in response to breast cancer cells (MDA-MB231) or colon cancer cells (SW480). Lenalidomide increased the frequency of Akt^+^ cells within the total CD3^+^CD8^+^ T cells or specific central (CM) and effector memory (EM) cell subsets, but not the terminal effector (TE) cell subset, by stimulation of XBP1-CTL with (B) breast cancer cells (MDA-MB231) or (C) colon cancer cells (SW480).

Understanding the mechanisms that influence the differentiation of CTL memory formation is critical for development of effective peptide-based cancer vaccines. Thus, we evaluated the impact of lenalidomide on activating the serine/threonine-specific protein kinase Akt, which acts as a critical signal integrator and plays a key role in orchestrating the activation and differentiation of memory CD8^+^ T cells. Our studies showed the specific upregulation in Akt expression within the CD45RO^+^ memory CD3^+^ CD8^+^ cell population of XBP1-CTL. Increased Akt expression was confined to the memory CTL, but not to non-memory CTL, upon stimulation with breast cancer (MDAMB231) or colon cancer (SW480) cells (Figure 8A). Lenalidomide induced Akt upregulation was seen in both central memory and effector memory XBP1-CTL subsets, but not the terminal effector subset, in response to breast cancer (MDA-MB231, Figure 8B) and colon cancer (SW480, Figure 8C) cells. Thus, these results demonstrate that lenalidomide treatment affects transcriptional regulators and the Akt signal integrator within memory CD3^+^ CD8^+^ T cell populations, resulting in enhanced anti-tumor activities of XBP1 antigen-specific CTL.

## Discussion

Following FDA approval of lenalidomide (Revlimid^®^) to treat patients with del 5q myelodysplastic syndrome and its use in combination with dexamethasone to treat MM patients, there are currently more than 220 clinical trials investigating its therapeutic role in solid tumors. As a single-agent, lenalidomide has been evaluated in patients with advanced brain, thyroid, hepatocellular, pancreatic, renal, breast, and colon cancer [27–29]. However, its ability to elicit tumor-specific immunity, especially its immunomodulatory activity in conjunction with tumor-directed vaccines, has not been well demonstrated. Our studies were designed to evaluate the potential role of an IMiD to enhance tumor-specific activities of antigen-specific CTL. We specifically investigated the effect of lenalidomide on XBP1-specific memory CD8^+^ CTL against breast cancer, colon cancer and pancreatic cancer cells, which overexpress XBP1 unspliced and spliced antigens.

XBP1 is a critical transcription activator participating in the UPR, a cellular adaptive mechanism that occurs in majority of tumors, and it allows the tumor cell to survive under stress conditions by increasing its protein folding capacity [30,31]. XBP1 regulates a subset of ER-resident chaperone genes that are essential for protein folding and maturation; and its upregulation has been reported in various human solid tumors including breast cancer, hepatocellular carcinoma, pancreatic adenocarcinomas and colon cancer patients [32–38]. During ER stress, an ER transmembrane inositol-requiring enzyme 1 (IRE1) oligomerizes, autophosphorylates, and via its endoribonuclease activity excises a 26 nucleotide intron from unspliced XBP1 mRNA, resulting in production of a potent XBP1 transcription factor that regulates a distinct set of UPR target genes. XBP1 has been demonstrated in the setting of breast cancer, to be an estrogen-regulated gene strongly correlated with estrogen receptor alpha (ERα) expression [20,21,39]. A recent analysis of independent cohorts reported that patients with triple negative breast cancer (TNBC), a form of breast cancer in which tumor cells do not express the genes for estrogen receptor, progesterone receptor, and HER2, have a specific *XBP1* gene expression signature that is strongly associated with poor prognosis; and a demonstrated a tumorgenic function for XBP1 branch of the UPR in TNBC. Importantly, XBP1 pathway activation correlates with poor patient survival in TNBC, suggesting that its specific inhibition may enhance anti-tumor therapies and representing as alternative treatment strategies for this aggressive breast cancer subtype [19]. Additionally, XBP1 mRNA splicing processed by IRE1 causes a reading frame shift which is translated into a spliced form of XBP1 protein, which is an active transcription factor in various cancers. High spliced XBP1 expression is associated with increased tumor growth and poor patient survival [40–42]. Thus, targeting the UPR and its associated molecular components is an emerging novel anti-cancer approach with early success in clinical studies.

With the rationale of targeting XBP1 for selective cancer cell killing, we have previously shown that immunogenic heteroclitic XBP1 peptides can evoke antigen-specific CTL with anti-tumor activities against MM cells and various solid tumor cancers [18,24–26]. In the current studies, we investigated the immunomodulatory effects of lenalidomide on XBP1-specific CTL phenotype and anti-tumor activities. Consistent with our previous study [26], lenalidomide induces cellular activation (high CD69 and CD38), upregulates CD28 and CD40L co-stimulatory antigen expression, but not inhibitory CTLA-4 nor PD-1 molecules, suggesting its potential beneficial effect to enhance immune responses of XBP1-CTL against tumors. CTL phenotypic changes induced by lenalidomide treatment correlated with their enhanced anti-tumor capacity, including IFN-γ and granzyme B production against a variety of solid tumor cancers. In agreement with other studies [43,44], our results provide direct support for a combination therapy using lenalidomide to increase cancer vaccine efficacy through various mechanisms of action. Our results emphasize the impact of lenalidomide on antigen-specific central memory CD3^+^ CD8^+^ CTL and their tumor-specific immune responses. In contrast, these studies indicate that lenalidomide does not appear to directly affect the expression levels of the intracellular XBP1 target antigens, nor extracellular activation/inhibitory molecules on tumor cells. These observations are consistent with the effect of pomalidomide, another class of IMiD, on tumor cells, suggesting that IMiD treatment has a better potential for modifying effector T cell function than directly influencing antigen expression on tumor cells.

The transcription factors T-bet and Eomes promote the generation and function of effector and memory CD8^+^ T cells via up-regulation of perforin/granzyme B during the early stages of CD8^+^ T cell activation, and by inducing migration to inflamed tissues through up-regulation of key chemokine receptors [45–47]. Studies have also demonstrated the contribution of these transcription regulators on anti-tumor immunity by showing that deletion of T-bet and Eomes in CD8^+^ T cells abrogate the persistence of effector and memory T cells [48]. Furthermore, clinical evidence showed longer patient survival with increased CD8^+^ T cell expression of T-bet and Eomes [49–51]. A melanoma-specific peptide vaccine trial showed high levels of T-bet expression in vaccine-induced CD8^+^ CTL, indicating that T-bet expression may be associated with CTL tumor infiltration and anti-tumor activities [52]. Results presented in these studies are consistent with our previous report of increased T-bet and Eomes expression in antigen-specific CTL generated *ex vivo* by immunogenic peptides stimulation [18]. Of particular importance in this study is the role of lenalidomide to enhance T-bet and Eomes expression, and the subsequent increase in functional anti-tumor activities of the effector cells. Specifically, we found that upregulated T-bet and Eomes expression following lenalidomide treatment of XBP1-CTL was mainly confined to the CD45RO^+^ memory T cell population, with the highest increase observed within the CM CTL subset, which corresponded with higher levels of IFN-γ and granzyme B production and increased cell proliferation in response to the various solid tumor cells.

Successful targeted immunotherapy development requires a better understanding of the signaling pathways that regulate quantity and quality of memory CD8^+^ T cell development, and the subsequent CTL anti-tumor activities. The serine-threonine kinase Akt has a role as a critical signal integrator that links distinct facets of antigen-specific CTL differentiation to specific signaling pathways of FOXO, mTOR and Wnt/β-catenin [53–55]. To date, how lenalidomide impacts Akt activation on effector T cell function, especially within antigenspecific CTL, is not well characterized. Thus, we investigated its effect on Akt activation within the XBP1 antigen specific-CTL. Our results demonstrated that enhanced Akt expression induced by short-term lenalidomide treatment was confined to the central memory and effector memory antigen-specific CTL subsets and corresponded with high level IFN-g production/granzyme B upregulation and T-bet/Eomes expression in response to tumor cells. The increased Akt activation by lenalidomide treated XBP1-CTL during tumor recognition may be associated with costimulatory signaling molecule CD28, a critical regulator of T cell proliferation and important for T cell activation and effector cells development [56,57]. However, sustained Akt activation might drive differentiation towards terminal effectors [58]; therefore we will investigate this aspect in future studies. Additionally, further studies using lenalidomide during CTL development are required to delineate transcriptional regulators involved, not only in generation of CD8 CTL, but also for long-term maintenance of memory/effector T cells.

In conclusion, we report on the critical role of lenalidomide to enhance anti-tumor activities (IFN-γ production, granzyme B upregulation, memory CTL proliferation) of XBP1 antigen-specific CTL through upregulation of key T cell activation markers/costimulatory molecules and downregulation of inhibitory molecules, which were associated with induction of Th1-specific transcription regulators and a signal integrator. Importantly, short-term lenalidomide exposure further increased the proportion of CD45RO^+^CCR7^+^ central memory CD3^+^CD8^+^ T cell subset within the XBP1-CTL, leading to enhanced anti-tumor activities against breast cancer, colon cancer and pancreatic cancer cells. These data provide the framework for a combination immunotherapeutic strategy encompassing both a cancer vaccine and IMiD to both enhance vaccine efficacy and promote long-lasting immune responses against cancer.
